# Community-Based Approaches to Reducing Health Inequities and Fostering Environmental Justice through Global Youth-Engaged Citizen Science

**DOI:** 10.3390/ijerph18030892

**Published:** 2021-01-21

**Authors:** Abby C. King, Feyisayo A. Odunitan-Wayas, Moushumi Chaudhury, Maria Alejandra Rubio, Michael Baiocchi, Tracy Kolbe-Alexander, Felipe Montes, Ann Banchoff, Olga Lucia Sarmiento, Katarina Bälter, Erica Hinckson, Sebastien Chastin, Estelle V. Lambert, Silvia A. González, Ana María Guerra, Peter Gelius, Caroline Zha, Chethan Sarabu, Pooja A. Kakar, Praveena Fernes, Lisa G. Rosas, Sandra J. Winter, Elizabeth McClain, Paul A. Gardiner, on behalf of the Our Voice Global Citizen Science Research Network

**Affiliations:** 1 Departments of Epidemiology & Population Health and Medicine, Stanford University School of Medicine, Stanford, CA 94305, USA; baiocchi@stanford.edu (M.B.); lgrosas@stanford.edu (L.G.R.); 2 Stanford Prevention Research Center, Department of Medicine, Stanford University School of Medicine, Stanford, CA 94305, USA; banchoff@stanford.edu (A.B.); jczha@stanford.edu (C.Z.); sjwinter@stanford.edu (S.J.W.); 3 School of Health, Care and Social Welfare, Department of Public Health Sciences, Mälardalen University, Box 883, 721 23 Västerås, Sweden; katarina.balter@mdh.se; 4 Health through Physical Activity, Lifestyle and Sport Research Centre (HPALS), Division of Exercise Science and Sports Medicine, Department of Human Biology, Faculty of Health Sciences, University of Cape Town, Cape Town 7725, South Africa; feyi.odunitan-wayas@uct.ac.za (F.A.O.-W.); vicki.Lambert@uct.ac.za (E.V.L.); 5 School of Sport and Recreation, Faculty of Health and Environmental Sciences, Auckland University of Technology, Auckland 92006, New Zealand; moushumi.chaudhury@aut.ac.nz (M.C.); erica.hinckson@aut.ac.nz (E.H.); 6 School of Medicine, Universidad de los Andes, 111711 Bogotá, Colombia; ma.rubior@uniandes.edu.co (M.A.R.); osarmien@uniandes.edu.co (O.L.S.); sa.gonzalez68@uniandes.edu.co (S.A.G.); 7 School of Health & Well Being, University of Southern Queensland, Ipswich, QLD 4305, Australia; tracy.kolbe-alexander@usq.edu.au; 8 Department of Industrial Engineering, Universidad de los Andes, 111711 Bogotá, Colombia; fel-mont@uniandes.edu.co (F.M.); am.guerra10@uniandes.edu.co (A.M.G.); 9 Department of Medical Epidemiology and Biostatistics, Karolinska Institute, 17177 Stockholm, Sweden; 10 School of Health and Life Sciences, Glasgow Caledonian University, Cowcaddens Road, Glasgow G4 0BA, UK; sebastien.chastin@gcu.ac.uk; 11 Department of Sport Science and Sport, Friedrich-Alexander-Universität Erlangen-Nürnberg, 91058 Erlangen, Germany; peter.gelius@fau.de; 12 Department of Pediatrics, Stanford University School of Medicine, Stanford, CA 94305, USA; chethan.sarabu@stanford.edu (C.S.); pkakar@stanford.edu (P.A.K.); 13 Gardner Packard Children’s Health Center, Atherton, CA 94027, USA; 14 School of Oriental and African Studies (SOAS), University of London, Bloomsbury, London WC1H 0XG, UK; praveenakfernes@gmail.com; 15 Research Institute, Health and Wellness Center, Arkansas Colleges of Health Education, Fort Smith, AR 72901, USA; elizabeth.mcclain@acheedu.org; 16 Faculty of Medicine, The University of Queensland, Brisbane, QLD 4072, Australia; p.gardiner@uq.edu.au

**Keywords:** health inequities, community-based, citizen science, participatory research, youth, health promotion, health equity, digital health, built environment, environmental justice

## Abstract

Growing socioeconomic and structural disparities within and between nations have created unprecedented health inequities that have been felt most keenly among the world’s youth. While policy approaches can help to mitigate such inequities, they are often challenging to enact in under-resourced and marginalized communities. Community-engaged participatory action research provides an alternative or complementary means for addressing the physical and social environmental contexts that can impact health inequities. The purpose of this article is to describe the application of a particular form of technology-enabled participatory action research, called the *Our Voice* citizen science research model, with youth. An overview of 20 *Our Voice* studies occurring across five continents indicates that youth and young adults from varied backgrounds and with interests in diverse issues affecting their communities can participate successfully in multiple contributory research processes, including those representing the full scientific endeavor. These activities can, in turn, lead to changes in physical and social environments of relevance to health, wellbeing, and, at times, climate stabilization. The article ends with future directions for the advancement of this type of community-engaged citizen science among young people across the socioeconomic spectrum.

## 1. Introduction

“*The future promise of any nation can be directly measured by the present prospects of its youth.*” John F. Kennedy

The continuing expansion of the world’s population has been accompanied by a parallel growth in health disparities, i.e., the unequal distribution of disease and associated risk factors both within and across nations, which increasingly have been recognized as inequitable [1]. Such health inequities have been driven by societal inequities across multiple levels and determinants of health, including individual, structural, socioeconomic, and environmental factors [2,3]. In light of the accumulating negative health impacts of such unfavorable conditions across a person’s life, the key importance of intervening at early life stages in addressing such factors has been increasingly recognized [4,5], as has the utility of targeting interventions at the community level [5,6].

The iatrogenic impacts of inequitable community structures and circumstances on the health and welfare of the world’s children and young adults living in under-resourced and marginalized communities have been well described [6,7]. They include a plethora of negative physical health effects and a range of behavioral and psychosocial impacts, including food insecurity, physical inactivity, and high levels of disengagement and disempowerment. Disengagement has been associated with participation in risky health behaviors (e.g., sexual activities, substance use, gambling) [8,9], worsened mental health (e.g., depressive symptoms, anxiety, suicidal ideation, feelings of helplessness/hopelessness and low efficacy for being able to change one’s circumstances) [8,10,11], and reduced participation in and increased drop-out from school [8,12,13]. Today’s young people also face the increasingly detrimental effects of global climate change, which poses threats to the planet as well as human health and welfare on a heretofore unimaginable scale [14]. 

Improving health, environmental, social, educational, and economic opportunities for young people across the socioeconomic continuum amidst a world struggling with the global impacts of climate change demands strategies that complement “top-down” system and policy changes with “bottom-up” community-driven approaches [15]. The participatory action research field has emerged over decades in recognition of the important contributions that such community engagement approaches can have in tackling the important social issues facing many communities [15,16,17,18]. Clinical fields such as nursing, for example, have used the Community Assessment, Intervention, and Empowerment Model (MAIEC) [19]. Yet a critical gap in knowledge remains concerning the availability of systematic, evidence-based and scalable participatory methods that engage and empower residents, irrespective of age and circumstances, not only to contribute to data collection and information transfer in these areas, but to become part of the solution to the social problems they face. This knowledge gap is especially glaring for adolescents and young adults —a complex and challenging developmental period [10,15,16,20,21]. The research presented in this article aims to address this specific gap by leveraging the power and attraction of mobile information and communication technologies (ICT) within a participatory action, “citizen science” framework for the youth and young adult age groups. Citizen science generally refers to nonscientists participating in the research process [22]. As part of this research, we use the traditional definition of “citizens” as inhabitants of a particular locality (without regard to legal status). The citizen science approach, which is part of the broader participatory action field, typically has included systematic, scalable methods of resident-based data collection to support the scientific endeavor [22]. Such methods have been aimed increasingly at youth in the biological, natural, and conservation science fields [23], though often have been separate from health issues facing residents and communities.

The accelerating growth of increasingly affordable mobile ICT provides a heretofore unparalleled opportunity to directly connect low-income youth to their local environments and communities in ways that can educate as well as inspire them to remain in school [12,24], and to positively engage with other important community structures and institutions that can impact health and local environments [25]. In addition, directly engaging youth from underserved and disadvantaged communities in meaningful ICT-enabled data-driven activities can allow them to become drivers, as opposed to bystanders, in shaping their local contexts in ways that can positively impact their own agency for improving their lives and communities [12,16,25].

The major goal of this article is to summarize the global youth-focused research projects to date that have employed a particular method of technology-enabled, community-based citizen science called *Our Voice* to promote health equity. *Our Voice* utilizes a “by the people” citizen science approach in which community residents and facilitating local community organizations are engaged in multiple contributory research processes that represent the full scientific endeavor. These processes can include development of study questions and/or objectives, data collection and interpretation, and formulation and implementation of relevant actions based on the results [22]. This approach contrasts with the “with the people” citizen science approaches typically used in other forms of citizen science, including those often used in the natural, ecological, and biological sciences (e.g., local bird counts, astronomy), where residents participate in systematic data collection but often not in other research processes [22]. The article ends with recommendations for next steps in applying this “by the people” citizen science approach to support youth in gathering and using their own data to help address health inequities and environmental injustice. Engagement in such participatory action approaches, when made relevant and accessible, may be especially beneficial for youth and young adults living in disadvantaged or marginalized circumstances [26].

## 2. Methods and Materials for the *Our Voice* Citizen Science Engagement Model

### 2.1. Overview

The *Our Voice* citizen science research model is an evidence-based and scalable “bottom-up” research-to-action model that engages and empowers residents as agents of change in their communities by giving them the tools to gather and deploy their own data to drive local change [22]. A major aim of this model has been to advance toward a state of health equity, whereby everyone has a fair and just opportunity to live the healthiest life possible [27]. By providing traditionally underserved community members with a voice in decision-making related to their local neighborhoods and communities, this model can complement “top-down” policy initiatives in a local area. *Our Voice* combines the strengths of traditional forms of community-based participatory research, including full involvement of community members in the scientific process, with community-based participatory design [28] and citizen science, which emphasizes more rigorous standardization of resident data collection methods than is often used in many forms of participatory research [29].

**Figure 1:**
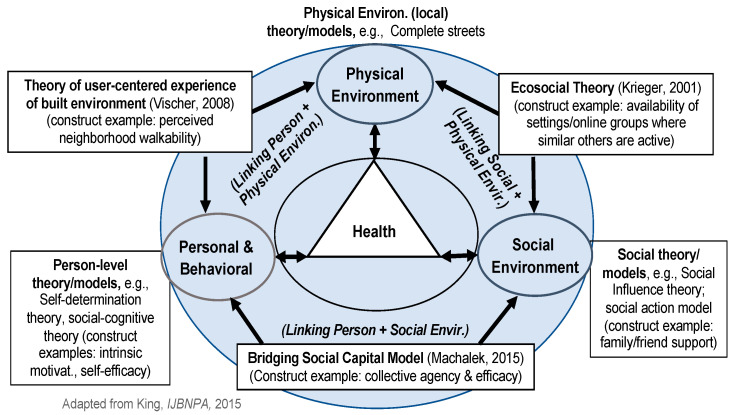
Dynamic theoretical approach to the socio-ecological model of health behavior change.

The model has been described in detail elsewhere [22,29], and is briefly described here. *Our Voice* sits within a dynamic socio-ecological framework of interconnected impacts, spanning person- to policy-level outcomes [30,31] (see Figure 1). It is informed by behavioral, social, and environmental theories and models of change (e.g., social cognitive theory [32], self-determination theory [33], social action models of community engagement [34,35]), complete streets perspectives), as well as by theoretical perspectives that traverse several levels of impact (e.g., eco-social perspectives that link physical and social environments [36]; bridging social capital models that link personal and social environmental levels [37]; the theory of a user-centered experience of the built environment, which links personal and physical environmental levels [38]). In addition, implementation science theory aimed at maximal scalability (e.g., the Reach Effectiveness Adoption Implementation and Maintenance (RE-AIM) framework; the Consolidated Framework for Implementation Research (CFIR) model to inform program implementation) can be brought to bear in exploring the model’s translatability across different populations and circumstances [39,40].

**Figure 2:**
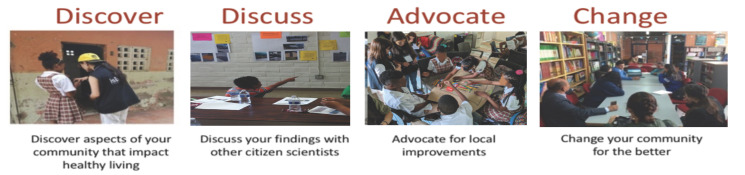
The 4-step *Our Voice* citizen science model. © Stanford University. All rights reserved.

The 4-step *Our Voice* model, shown in Figure 2, begins with an easy-to-use, multi-lingual, mobile app, called the Stanford Healthy Neighborhood Discovery Tool^TM^ [41], as the “gateway” to the community research process [22,29]. Remotely trained local community facilitators support *Our Voice* citizen scientists in using the app to gather relevant geo-tagged data consisting primarily of photos, audio and/or text narratives, and ratings of local environmental features that help or hinder specific health-promoting behaviors or conditions (e.g., walkability, food access, safety, well-being, safe transit to school, access to recreational areas for play and physical activity). The citizen scientists are then prompted to upload the multi-dimensional, anonymized data to a secure server at Stanford, where collective data reports are generated and then returned to the community for discussion and use. The Discovery Tool secure data repository has been approved by the Stanford University Institutional Review Board (IRB) for the protection of human subjects (IRB protocol #40379). Collaborating research organizations also obtain human subjects/ethics approval from their respective academic institutions. Non-academic partners collaborate with Stanford under Stanford’s IRB protocol #45330.

Once the Discovery Tool data are returned to the community, the Stanford-trained and supported local community facilitators conduct several interactive meetings where the citizen scientists discuss, analyze, and interpret their collective data to identify and build consensus around high priority local issues to address. They then brainstorm the types of relevant local decision-makers and stakeholders who could be helpful in advancing proposed changes. Often the facilitators will invite decision-makers to a subsequent group meeting where the citizen scientists present their data and discuss potential solutions that could realistically be enacted. Agreed upon action steps are identified and subsequently monitored for change. While originally developed using in-person intervention delivery, the model has been successfully adapted for fully remote delivery in response to the COVID-19 pandemic.

The citizen science process implemented in the *Our Voice* model has been shown to be convenient and minimally burdensome to both community residents and participating local organizations [29,42]. Because the *Our Voice* process is typically focused on a particular health-related issue within a specific locale, suitable resident agreement around the major environmental enablers and barriers often can be attained with as few as 8–10 residents [43]. While documentation focuses on community assets as well as current challenges, the process has also been found to be acceptable and engaging for local decision makers and stakeholders [29]. Activities within each step of the 4-step sequence (summarized in Figure 2) can be customized as needed to meet the needs and constraints of a particular population or locale. All project leaders are encouraged to include relevant multi-level measures, as described in a previously published *Our Voice* Global Network scientific article [44], which can be shared across the *Our Voice* Global Network. Final determinations concerning the most relevant assessment battery for each project are made by each project group, with a small core set of putative intervention mediators relevant to this participatory action model collected in most projects [29]. These can include changes in the following variables: sense of civic engagement and responsibility; personal and collective empowerment and agency; advocacy skills; neighborhood cohesion and social networks; group communication and consensus-building skills; and technology and health literacy [29]. Typically, a mixed-methods measurement approach is taken, including anonymized qualitative, quantitative, and geospatial data collection, with the data integrated on the *Our Voice* data portal and returned to local project leads/facilitators to share with community members [29]. The proximal goals of the intervention are to drive changes in local physical and social environmental structures and activities that are tied directly to the specific issue targeted for action (e.g., increased walkability, enhanced safety, easier access to healthy food options). Through executing or sustaining such changes, the potential for more distal effects on health and wellbeing become increasingly likely, in light of the consistent associations found between local physical and social environments and a plethora of health and quality of life outcomes [45,46,47]. It has been noted that focusing on local environments may be particularly important given the contextual variability found across locales [48,49]. For example, an investigation of national US built environment data reported that while disadvantaged neighborhoods were in general more walkable, census tracks with a higher proportion of children and older adults were less walkable after adjusting for region and level of urbanicity [49]. In addition to facilitating changes in relevant local environmental structures or conditions, community participatory action models such as *Our Voice* can foster feelings of agency, collective efficacy, empowerment, and control among participants that can help to promote continued civic engagement and participation [3,18,29].

The *Our Voice* citizen science model itself has been applied or is currently being tested in over 20 countries across six continents, addressing an increasingly diverse range of local issues impacting health [29]. Early on, citizen science research initiatives such as *Our Voice* targeted primarily adults in diverse under-resourced communities around health and safety issues [42]. This has changed with the heightened appreciation of the benefits and impacts of involving youth directly in community-based participatory research [25]. Through such youth-focused participatory action projects, a growing number of which have leveraged the potential power and reach of evolving information technologies [25], it has become increasingly clear how compelling and persuasive such data-driven narratives, told through the eyes of young people, can be in fostering positive personal and community change to improve health, well-being, and social capital [50,51,52].

### 2.2. Description of Our Voice Research Projects Aimed at Youth and Young Adult Populations

**Figure 3:**
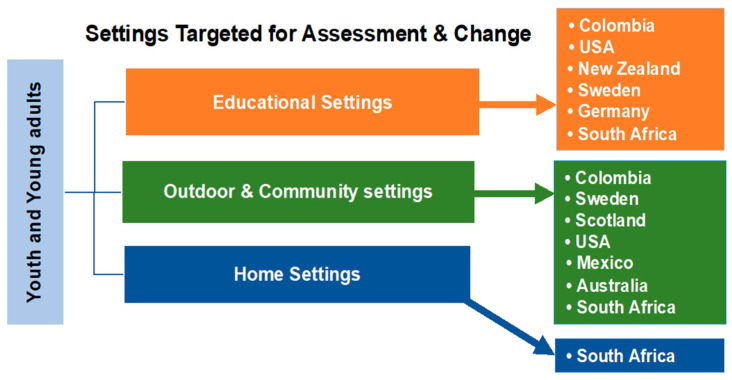
*Our Voice* youth and young adult projects.

As summarized in Figure 3, the youth and young adult *Our Voice* research projects that have been conducted to date or are in process have focused on three types of environmental settings: educational, outdoor/community, and home settings. While a number of projects have targeted one setting in particular, several have focused on multiple settings.

To place *Our Voice* projects within the broader field of participatory action research involving youth, we calculated the proportion of projects in which youth or young adults had direct decision making power or engagement, either as leads or as active collaborators or contributors, in each of nine participatory research processes [25,53]. For each of the 20 projects, the lead research team indicated whether the citizen scientists had or were in process of taking the lead or otherwise being directly involved in each research process. The nine participatory research processes were decision-making about the research question, designing the research and methods, identifying or preparing research instruments, identifying and recruiting participants, collecting data, analyzing data and drawing conclusions, producing reports or summaries, disseminating findings and reports, and advocating or mobilizing for policy impact [25].

## 3. Results

### 3.1. Summary of Our Voice Youth and Young Adult Projects Completed or in Process

**Figure 4:**
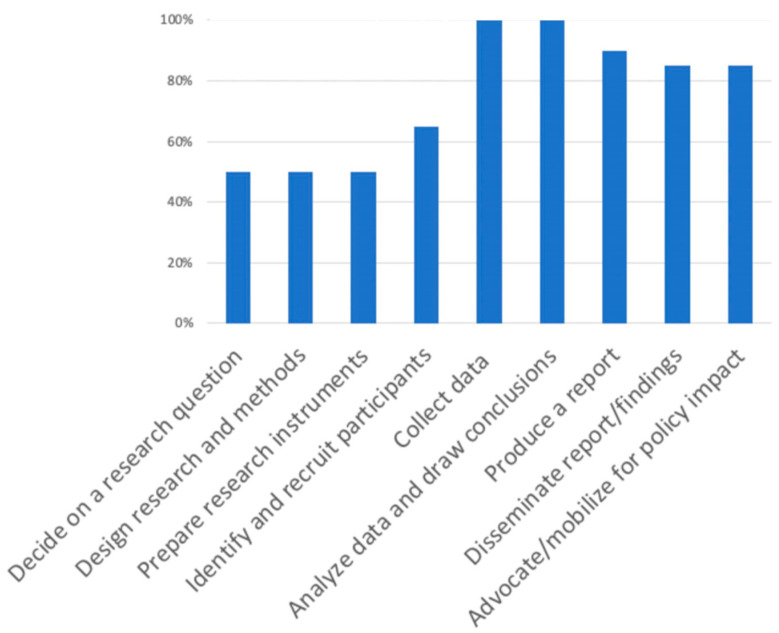
Proportion of participatory processes in which youth were directly engaged.

Thus far, 20 *Our Voice* research projects developed as part of the *Our Voice* Global Research Network and involving youth (typically ≤ 18 years of age) and young adults (typically between ages 18 and 24 years) have been completed or are in process around the world. All 20 projects have employed the *Our Voice* methods described earlier in collaboration with the Stanford *Our Voice* team to ensure the fidelity of the methods being used. Figure 4 summarizes the proportion of these projects in which young people have been engaged directly in each of the nine different participatory processes described earlier that are part of the participatory action research literature [25,53]. An age gradient was observed, with older individuals engaging in a larger number of processes relative to younger individuals. For example, all five of the projects involving young adults had citizen scientists engaging in all nine participatory processes. In contrast, of the 15 projects involving younger participants, only 27% engaged them in all nine participatory processes. As one might expect, projects involving younger children often involved less engagement in the initial three participatory processes typically requiring more advanced levels of conceptualization and knowledge, i.e., research question selection, research and methods design, and participant identification and recruitment. In contrast, all projects reported youth engagement in active data collection as well as data discussion/analysis and interpretation. Engagement in the latter participatory process of data discussion/analysis and interpretation differentiates this type of “by the people” citizen science method from the aforementioned “with the people” citizen science approaches [22]. In those latter types of citizen science, the data collected by residents are typically transferred to scientists or similar professional groups for subsequent review and interpretation. Over 80% of all *Our Voice* research projects described also engaged their citizen scientists directly in producing reports or summaries of what they learned (85% (n = 17)), disseminating their findings (90% (n = 18)), and advocating and mobilizing for policy impact (85% (n = 17)).

Table 1 summarizes all of the global *Our Voice* youth and young adult projects that have been completed along with examples of others that are currently in process. This overview table is organized by age groups of residents participating, i.e., intra-generational research projects involving only youth (i.e., ≤18 years of age) as citizen scientists, inter-generational research projects involving both youth and adults in citizen science activities, and college age/young adult research projects involving young adults only as citizen scientists.

### 3.2. Intra-Generational Youth Projects in Educational Settings

Among the many causes of global poverty, one factor stands out—education. At a population level, educational attainment has the ability to accelerate many of the solutions to poverty, among them economic growth and reduced income inequality, improved nutrition and preventive health, and reduced violence at home and in the community [54,55,56]. In addition to educational policies and systems that are key factors in educational attainment, a potentially powerful driver is the level of disempowerment and disengagement experienced among youth living in under-resourced and marginalized communities worldwide. This often manifests in lack of knowledge, resources, and personal and collective agency to modify factors in their social and physical environments, including the school environment, that can improve their own as well as their community’s welfare [16,56,57]. As noted earlier, among the negative ramifications of such disempowerment and disengagement during adolescence and young adulthood are participation in risky health behaviors (e.g., substance use, gambling) [8,9] decreased mental health [8,10,11], and reduced participation in and increased drop-out from school [8,12,13]. School settings therefore represent a ubiquitous and highly relevant location for teaching youth about participatory action research methods that may improve their local school environments while also providing a means for actively engaging them in positive change.

*Our Voice* citizen science school projects have occurred in several different locations in Colombia (Table 1), where students ranging in age from 9 to 18 years and living in low-income neighborhoods have collected data, using the Discovery Tool mobile app, both within the school setting itself as well in the area surrounding the school. They then have been able to build consensus around high priority issues affecting their health and safety in the school environment, and successfully advocated for realistic changes. Among the changes occurring from these efforts have been healthy school initiatives aimed at improved food options at school, increased infrastructure maintenance, improved safety measures related to automobile traffic around the school, efforts to reduce other hazards such as stagnant water, and students’ acknowledgement of their co-responsibility for properly using and maintaining their school’s infrastructure [58,59].

In an intermediate school in Auckland, New Zealand, students ages 10–13 years have been working with the school principal and senior leadership to address some of the barriers to a healthy and safe school environment identified during their data collection process. Barriers identified by the students included vandalism, lack of fencing around the school grounds to promote a feeling of safety, litter on the school grounds, and a need for the playground equipment to be upgraded. The student advocacy groups have presented their results to their school (through a special assembly), the Board of Trustees and principal, as well as at the 2019 SouthSci-sponsored Project Symposium (the project funder) [60].

School-based programs with youth also have been actively engaged in promoting healthier environments outside of the school facility itself. For example, in a U.S. citizen science project with ethnically diverse intermediate/middle school children ages 12 and 13 years, the major focus was on identifying factors helping or hindering safer walking and biking routes to school [61]. The students identified a number of barriers to walking or biking safely to school, including traffic congestion around the school, lack of pedestrian crosswalks and traffic signage, broken sidewalks, and lack of bike racks. The students were able to enact new peer-to-peer training activities across the school year (e.g., appropriate helmet use) and present their data and recommendations to stakeholders from nine different sectors [61]. While plans were made with the City public works engineer to modify car and bicycle entries and exits at the school to improve safety, the subsequent departure of the engineer resulted in this plan being delayed. Such occurrences underscore the importance of including a range of potentially relevant solutions to the challenges being identified, given that stakeholder involvement may change over time. The active transportation goals focused on in this project additionally have been linked regularly to environmental sustainability and climate stabilization objectives worldwide, including air quality and carbon footprint goals [62,63].

Another example of a school-based youth program that went beyond the school environment itself was conducted in Glasgow, Scotland [64]. The elementary school students in an environmental sustainability after-school club participating in this project (ages 8–10 years) came from among the most economically deprived areas in Scotland and focused on the canal network crossing these areas. This canal network was closed and in derelict condition until 2001. It is currently being regenerated with the aim of offering the local communities a place for recreation and flood defenses. After conducting Discovery Tool-based data collection, the students discussed the positive and negative features of the canal area, and then independently developed an animated film describing their data-based results and recommendations, which was shown to the school and other local authorities. Their results and recommendations were also integrated into a larger report which was shared with the Scottish government. These citizen science activities contributed to efforts facilitating the subsequent building of a new multi-use outdoor game space for youth at the canal site as well as a bridge linking three communities previously physically separated by the canals.

Additional *Our Voice* citizen science projects are underway that use schools as the jumping off point to capture information about the school environment as well as other local environments in which youth live, travel, and play. A citizen science project in Cape Town, South Africa is investigating the socioecological levers that impact diet and physical activity behaviors in South African adolescents across their entire day [65]. A total of 30 adolescents in Grade 8–11 have been recruited as citizen scientists from three urban high schools in Cape Town which vary in terms of being in low-income vs. middle- to higher-income neighborhoods. The recruited citizen scientists represent students of all genders who live and go to school in the same area or, in some cases, live in a low-income neighborhood but travel and attend school in a middle-to-higher income area. Student volunteers include those who are recognized peer leaders (e.g., members of the schools’ Representative Councils of Learners), as well as adolescents without any specific school leadership roles. They will collect data using the Discovery Tool mobile app to capture factors that influence their food choice decisions and physical activity within their school, during the journey from home to school, at home, and in their neighborhood environments. The multi-environmental focus of this research will shed light on the interrelations between the different contexts that together influence adolescents’ activities and health behaviors throughout the day. Following data collection, the citizen scientists will attend a workshop to analyze and discuss their data in order to identify and prioritize potential and mutable barriers to improving access to healthy, affordable food and opportunities for physical activity in the different environments being evaluated. Although this project had to be halted during data collection due to the COVID-19 pandemic, it is planned to be resumed as soon as it is safe to do so. Preliminary results thus far collected via telephone interviews with low-socioeconomic status (SES ) youth and focused on their home environments suggest that youth perceive a lack of autonomy in food choices at home and did not experience support from their families to participate in physical activity, which often was considered to be unsafe [66]. The regular physical activity that they did receive came from the chores that they did at home. The area in which they exerted the most autonomy was in their food choice purchases from local shops using their “pocket” money. These purchases were typically calorie-dense, nutrient poor snack foods [66].

The Cape Town project is the first *Our Voice* youth project to directly target the home environment. Home environments are a particularly important setting for youth health and well-being but have received generally less attention in the citizen science health arena than other settings. Given that current COVID-19 policies and restrictions have forced many schools around the globe to enact remote learning or similar forms of distance learning for many students and classes, understanding both the barriers and enablers of learning in the home setting have become especially important. For example, researchers in Colombia have observed that many children may not have adequate locations in their home environments to complete homework assignments or undertake remote learning. Increasing our understanding of the most cost-efficient and productive ways for facilitating remote learning in diverse home settings is particularly indicated during such challenging times and could likely have important spill-over effects with respect to youth and family health and well-being.

### 3.3. Young Adult Projects in Educational Settings

The growing number of *Our Voice* citizen science research projects engaging students in university and college settings reflects the particular interest in and relevance of such participatory action projects for this age group [53]. As noted earlier, individuals in this age group are typically able to engage directly in all nine participatory processes described in this literature [25,53]. The issues that have been targeted for study have spanned a diverse set of problem areas. For example, an *Our Voice* project on a U.S. university campus has been the first to evaluate the utility of applying this participatory model to the gender-based violence field–a frequent and growing issue facing many campuses in the U.S. and elsewhere [67]. In this formative study, 10 undergraduate women aged 18–20 years used the Discovery Tool mobile app to identify social spaces and situations associated with feeling uncomfortable and unsafe or comfortable and at ease based on one’s gender [68]. Among the situations that were rated as uncomfortable were shared-gender toilets, noisy or raucous Greek fraternity parties, and workspaces, gyms, and other settings in which men predominated. Among the strategies proposed during group citizen scientist discussions were simple modifications to bathrooms in dormitories to facilitate more privacy, increasing the number of nonfraternity-controlled campus parties, and hiring more women faculty and teaching assistants. The citizen scientists identified key decision-makers that could help to facilitate such changes, have summarized the results for publication, and are in the process of developing additional ways to further disseminate the findings in laying the groundwork for developing specific actions for change. The project aims to move to the final phases of the model which include citizen scientist presentation of results to student and university leadership groups. The aim is to follow this by collaborative advocacy and action determinations for changes aimed at promoting safety and diminishing gender-based violence on campus. Perceptions of diminished safety in these and other community environments have been associated with a range of negative health behaviors as well as health outcomes such as higher body weight [69].

An *Our Voice* citizen science research project with university students from four university campuses in Auckland, New Zealand has targeted campus-based built environment features influencing student physical activity and eating patterns [70,71]. Eighty-one students from ethnic- and age-diverse backgrounds across the four university campuses participated as citizen scientists. Among the issues identified as important as well as feasible for change were limited access to affordable healthy food options onsite and in vending machines, limited availability of water fountains, lack of gymnasium visibility and awareness, and unused green space that could be used for physical activity. In the next phase of this research, students will present their findings to the student union and senior leadership of the university and advocate for realistic changes to the food and physical activity environments on campus.

Other university citizen science projects have included those aimed at promoting gender equity on campus as well as fostering mental health among graduate students, including during the COVID-19 pandemic [72] (Table 1). In an *Our Voice* project being developed for implementation on a college campus in Arkansas, osteopathic medical students will be invited to participate in a collaborative project where they will be paired with local high school students to explore how access and equity impact health disparities. Following completion of *Our Voice* training sessions, the student pairs will investigate community neighborhoods and document images that promote or hinder walkability and safety. They will utilize the Discovery Tool app to document their observations and complete guided thematic analysis of their data. They then will apply the findings to gain a realistic experience of how the social determinants of health can impact access, equity, and health disparities. Students will use their findings to inform ongoing and future quality improvement-oriented health promotion/disease prevention projects at the community and public-school levels.

In addition to conducting projects involving the full *Our Voice* 4-step model, a group of university faculty in Germany have utilized the Discovery Tool mobile app technology as a teaching tool in an international graduate student course introducing students to different scientific assessment methods. Students employed the Discovery Tool app to obtain hands-on experience with a qualitative assessment method, and then reported on what they found to be both positive and negative aspects of this type of method. The students collectively completed 40 Discovery Tool walks and recorded 359 photos as part of a class assignment to explore neighborhoods with a focus on opportunities for and barriers to physical activity. The students reported that the Discovery Tool was generally easy and convenient to use and provided them with insights related to implementing such technology-enabled qualitative assessment methods for capturing important aspects of local built environments. In addition, students provided feedback to improve the German translation of the Discovery Tool. Other college coursework on U.S. campuses employing the Discovery Tool have included courses in health areas such as obesity and health-related citizen science, student independent research aimed at systematically capturing the lived experiences of young female athletes, and factors influencing community health among young adults living in Algeria. Additionally, students from the Arizona State University College of Health Solutions have been documenting, as part of their coursework, environmental health indicators in downtown Phoenix, Arizona which they plan to share with local policy makers [73].

### 3.4. Inter-Generational Youth Projects in Educational Settings

In addition to intra-generational projects involving middle- and high-school students, at least one *Our Voice* project aimed at elementary school environments has involved parents as citizen scientists in capturing local features that enable or hinder active transport (walking, biking) to school [61]. This study, aimed at an ethnically diverse school district in northern California, USA, used a prospective controlled comparison design to compare the increases in walk/bike rates to school in two elementary schools that were receiving the federally supported Safe Routes to School (SRTS) program for the first time (Table 1). The school receiving the SRTS program augmented with the *Our Voice* intervention engaged in twice as many SRTS educational and engagement activities across the school year compared to the school receiving the SRTS program alone [61]. In addition, based on independently collected standardized tallies of walking/biking to school at the end of the school year, the *Our Voice* school’s walk-bike to school rates were twice as high as the comparison school (*p* < 0.001) [61]. The increased SRTS school-wide engagement activities in the *Our Voice* school continued into the following school year, during which time that school had a three-fold greater number of such activities relative to the comparison school [61]. Dissemination of project results through media articles has led a County Department of Education to partner with local parents to initiate the model in a large school district in northern California. Expansion of such projects could positively contribute to environmental sustainability and climate stabilization measures in diverse communities around the world.

Another project will use *Our Voice* to address the lived experience of food insecurity and health beliefs in an ethnically diverse western Arkansas community [74]. The project will document the impacts of nutrition education and health coaching on health nutrition behaviors, and development of a culture of health among middle school students and their families. The project will match two middle schools from the same public school district by ethnic diversity, school size and Supplemental Nutrition Assistance Program (SNAP) annual reporting. 15–20 students ages 12–15 years with parent or guardian participation from each school will be invited to participate as citizen scientists. One school group will receive a focused nutrition curriculum, hands-on learning and health coaching in the fall semester. The second school group will receive the intervention in the spring semester. All participants will receive training on the Discovery Tool app. All participants will document health behaviors, including healthy or unhealthy nutrition choices and access or lack of access to nutritious food in their community. Students and families will be guided through the group process to identify themes in the fall and again in the spring. Qualitative inquiry and quantitative analysis will be used to assess individual and comparative analysis of school themes at the end of the academic fall and spring semesters. Analyses will explore experiences, change and sustainability of health beliefs and behaviors surrounding nutrition and food insecurity and assess the impacts of the curriculum on behavior change associated with the interventions. All results will be incorporated in the development of community-informed, targeted initiatives/interventions to improve health behaviors and address food insecurity in building a health community.

Finally, an inter-generational *Our Voice* citizen science project is being launched in Toowoomba, Australia with 10 to 11-year-old children and 18–24-year-old young adults that is aimed at engaging young people in data collection and solution-building targeted at the Toowoomba Regional Council’s identified goal of encouraging more active transport and use of public transportation in the Toowoomba Region. The Council’s data indicate that nine out of every ten trips in Toowoomba are made with a private vehicle, despite the average distance per trip being less than five km. These private vehicle transportation rates have been found to be higher in Toowoomba than in surrounding areas of Queensland, Australia. Young people will participate in the full *Our Voice* 4-step method [22], with the goal of generating and helping to enact realistic solutions for increasing active transport among these two age groups as well as others in the community.

### 3.5. Intra-Generational Youth and Young Adult Projects in Outdoor and Community Settings

A growing number of *Our Voice* projects have focused on outdoor environments that include neighborhoods and other community settings as important targets for promoting health-enhancing change. Such intra-generational youth projects typically involve adolescents. One such project, undertaken with multi-ethnic adolescents from low-income neighborhoods, was conducted in Sweden [75,76]. Twenty-four adolescents from two low socioeconomic status (SES) neighborhoods used the Discovery Tool app to identify local environmental features that fostered or hindered physical activity. Among factors impeding regular physical activity were diminished walkability and bike-ability infrastructure, limited access to public sports facilities, and neighborhood safety factors, including poor lighting in an open area at the center of one of the neighborhoods which had become a gathering spot for male adolescents. This in turn has led female adolescents to feel uncomfortable walking near that area. The findings are being used to apprise local policy makers and stakeholders of ways to improve these neighborhoods to promote more physical activity among young people. They also underscore the types of gender-specific perceptions of local environments, noted as well in the gender-based violence study described above, that deserve further investigation.

A second *Our Voice* citizen science study is being launched with Swedish adolescents from disadvantaged neighborhoods to systematically explore the relationship between meaningful leisure time activities and how such activities may affect high school completion rates. Approximately 20 adolescents ages 16–19 years will be recruited from a local youth center and will collect data using the Discovery Tool to identify local positive and negative activities and features that contribute to meaningful leisure time and support adolescents’ ability to graduate from high school. These data will then be processed, discussed, and prioritized by the adolescents using a facilitated group process, with the results being used to help guide future initiatives and the work of local agencies and community settings (e.g., schools, youth centers, libraries, places of worship) aimed at keeping adolescents from disadvantaged communities in school. Keeping adolescents from dropping out of school has been recognized as an important driver of global poverty and economic inequality [54,57]. As noted in the United Nations Sustainable Goals Report [77], if all persons completed secondary education, the global poverty rate could be cut by more than half. As noted earlier, a potentially powerful driver of poor educational attainment includes the negative impacts that levels of disempowerment and disengagement experienced by young people in under-resourced and marginalized communities worldwide can have on school performance and drop-out [8,12,13]. The type of youth-engaged participatory action research being described represents a systematic community-engaged “bottom-up” method that can be added to broader “top-down” system and policy approaches for improving educational attainment outcomes in disadvantaged communities around the world.

### 3.6. Inter-Generational Projects in Outdoor and Community Settings

In addition to those research projects aimed at outdoor and community settings which have targeted young people only as citizen scientists, a number of *Our Voice* projects have engaged multiple generations in shared citizen science and advocacy activities. Among the inter-generational projects that have been completed are a collaborative project between a grassroots residents organization (SOMOS Mayfair) and the local public health department in a lower-income neighborhood of San Jose, California which created better access to local parks and recreational spaces for all residents [29]; an intergenerational project in Cuernavaca, Mexico to support active living across socioeconomic strata which helped to increase intergenerational communication and understanding related to graffiti and public art [78]; a project in a Jerusalem, Israel neighborhood involving 23 older adults and 15 high school students which promoted building a shared vision for healthy living for all ages [29]; and an intergenerational project targeting neighborhood walkability, aesthetics, and security in a primarily Latinx neighborhood in northern California which improved practices related to illegal dumping and trash occurring in this low-income neighborhood from surrounding, more affluent neighborhoods [79]. In this Latinx neighborhood project, the youth participants were particularly concerned with how illegal dumping and trash in their neighborhood could be carried into the San Francisco Bay during the rainy season, disrupting the fragile ecosystem and animal and plant life in the Bay [79]. Their discussions emphasized this as an environmental justice issue that created concern and anxiety.

#### Addressing Disparities beyond Clinic Walls through Clinic-Community Citizen Science

A recently initiated *Our Voice* citizen science project in the San Francisco Bay area, California focuses for the first time on community health clinics. The clinics being targeted serve families from low-income, underserved communities, a number of them of Latinx descent. The focus on health clinics is based on the premise that, while there is compelling scientific evidence that individual and community health are shaped by the local environmental and social contexts in which people live, work, play, and learn [6], clinical interactions rarely account for such contexts. Given that traditional “top-down” policy approaches to changing the “upstream” determinants of health often are inaccessible to low-income and underserved communities, the frequent result is deepening health disparities across broad groups [6,17]. The COVID-19 pandemic has exacerbated such health disparities while transforming human behaviors and interactions—both in clinical contexts and in public spaces—in paradigm-shifting and often unhealthy ways. This is particularly true for low-income communities with limited resources to prevent and combat the catastrophic consequences of the pandemic. To address the resulting health disparities, it is critical to develop and test innovative, upstream participatory approaches that build capacity for preventive health behaviors while also promoting healthy environments in vulnerable communities. This project represents a novel, first-time adaptation of the *Our Voice* citizen science model for a community primary care clinic setting. The project aims to connect clinical providers with patients in a systematic process of documenting and activating feasible health-promoting changes in their local environments to enhance their health and well-being. It will do this through integrating healthcare providers and clinic teams with patients and researchers in practical ways throughout the *Our Voice* discovery/data collection, discussion, and action phases. Table 2, which was developed by one of the co-authors (Sarabu), summarizes examples of how clinical providers and staff could be integrated as part of the *Our Voice* model. The examples show how, through patient data collection, remote group discussions and community-level (privacy-protected) data sharing, clinics can become more community-embedded, community-responsive, and community-committed in new ways.

**Table 1:** Examples of *Our Voice* youth and young adult projects completed or in process.

		Community Features Identified		Strategies Proposed and Changes Enacted	
		Positive	Negative		
**Intra-Generational Youth Projects**				
Gilroy, CA (USA) Safe Routes to Middle School [61]	Ethnically diverse school children in grades 6 to 8 from Gilroy, CA (total city population = 53,231); (N = 26 children participated, ages 12–13 yrs., 70% girls)	Crossing guards Pedestrian signaling at certain intersections Presence of pedestrian trails & sidewalks	Traffic violations & congestion Safety concerns Lack of crosswalks Lack of pedestrian/cyclist education Lack of traffic signs Trash, broken sidewalks Lack of bike racks	14 Safe Routes school engagement/education and peer-to-peer training activities enacted across the school year (e.g., bike assembly, appropriate helmet wear & use) Students met with and presented their data & recommendations to 16 stakeholders from 9 different sectors Students invited to speak at a Youth for Environment & Sustainability Conference Advocacy & outreach to the City Bike & Pedestrian Commission Changes in car & bike entry and exit from school area planned with City Public Works engineer, but change in city personnel delayed execution Successful peer-to-peer training of next student cohort to continue to lead school-based Safe Routes to School activities
Bogotá, Colombia Building Healthy Schools through Technology-enabled Citizen Science [58]	5 urban public schools teaching grades elementary through high school (N = 97 youth participated, ages 9–18, mean age = 13.4 + 2.2 yrs., 64.9% girls)	Availability of sports facilities & equipment Environ. aesthetics/ green spaces Availability of school meal programs & water fountains	Poor maintenance of classrooms & bathrooms Unhealthy foods Higher cost of healthy foods Lack of civic culture to maintain a clean school environment	New healthy school initiatives occurred, including a Tree Planting day, initiation of healthier breakfasts at school, & awareness campaigns highlighting school improvements being made School policy makers committed to develop a proposal for private investment to improve maintenance of school facilities; connect students with District’s Education Dept. to increase direct communication; create action steps with each schools’ Wellness Coordinators to improve bathroom allocation & maintenance; encourage school participation in inter-sectoral local meetings coordinated by Bogota’s Environmental Dept. Students committed to taking better care of their school facilities & environment, and utilize their advocacy skills in making specific, realistic requests to the District’s Education Dept.
Barú, Colombia Enhancing Within-School environments [59]	1 rural public high school (N = 11 adolescents, ages 13–17, 73% girls)	Access to the library to relax and learn Existence of specific classrooms to carry out studies that related to technical or technological degrees Green spaces Specific clean and well-maintained areas Recreation spaces	Heavy traffic and high speeds on the main street in front of the school Danger of risks and personal injuries due to the condition of the infrastructure and the presence of waste in common areas Poor maintenance and availability of bathrooms Bad condition of chairs and tables Low-rise wall that allows students to escape from school Poor maintenance of common and recreation areas Poor condition of fans	The traffic police committed to assigning personnel to provide road behavior & safety training The Amor Por Barú Foundation, a local partner of the project, made arrangements in the green areas and in the parking lot in order to increase the safety and well-being of the students An architect worked on solving the stagnant water problem A new adult bathroom was built for teachers & staff Citizen scientists presented their findings at the school’s Youth Social Forum The ecological groups at the school were strengthened based on the findings of the citizen scientists The school adopted the methodology of *Our Voice* in additional projects that involved both elementary and high school students using their own devices and cameras The students organized clean-up campaigns in the areas they had identified The students organized sense of belonging campaigns to improve the care of school facilities The students organized ways to improve the school gardens; they spoke to local fishermen for help and wrote a letter to the most important hotel in the area to get compost soil and plants Students now participate more in collective initiatives and are more involved in participating in the student council A space for conversation with the principal was created to improve trust between directors and students Adolescents have begun to contact the village community council to become more involved in the problems of their community
Barú, Colombia Enhancing Neighborhood environments surrounding schools [59]	1 rural public high school (N = 12 adolescents, ages 13–17, 67% girls)	Rec. & sport spaces School as a source of learning Gastronomy (typical dishes of the region) Culture and environment (crafts) Roads in good condition	Environmental pollution Destroyed parks and recreation spaces Drug addiction Danger of personal injury Fights Noise pollution	Citizen scientists committed to increase the sense of belonging in the community by taking care of recreation and sports spaces Adolescents have begun to contact the village community council to become more involved in the problems of their community The school committed to sensitizing the community to not throwing garbage on the streets
Västerås, Sweden Capturing prerequisites for safe physical activity among low-SES adolescents [75,76]	Adolescents (N = 24) ages 16–19 (mean age = 16.6 ± 0.8 years, 75% females) from deprived neighborhoods	Parks, playgrounds, outdoor gym Other amenities Sport facilities Aesthetics	Vehicles; vacant lots Surveillance Construction work Public safety threats Poor public transport Deficient infrastructure for walking, biking Lack of lighting	Low-socioeconomic status (SES ) adolescents living in Swedish neighborhoods found the mobile app-based neighborhood audit tool to be a feasible and accessible method for capturing positive and negative features of their local environments for physical activity Plans by researchers to share findings with policy makers in Västerås and similar cities as a step towards developing realistic solutions
Auckland, NZ Empowering children to influ-ence changes in school environ-ments for learning, PA, health, wellbeing [60]	Intermediate (middle) school age children in Years 7 & 8 (10–13 years); N = 241	Sports fields—Help students keep active and play with friends Confidence course/playground Peace garden—Good area to clear your mind and relieve stress Food tech class—learn something unique	Vandalism: Graffiti, broken bathroom locks and lack of mirrors in girls & boys’ bathrooms Safety: Lack of fence around the school grounds Environment: litter around the school grounds and upgrade of playground needed	Student advocacy group presented to school’s principal and senior leadership team. The main issues raised where bathroom locks, mirrors and graffiti. The principal promised to fix the issues raised Academic team invited back in 2020 to review the changes Initiation of action group team from current Year 7 & 8 students to meet with teacher leads to discuss issues pertaining to school environment, with the goal of continuing to make realistic changes in the school environment.
Glasgow, Scotland Enhancing urban green and blue spaces [64]	Environmental afterschool club in most deprived area of Glasgow; children from 3 elementary schools (N = 18, 48% Girls, age 8–10) took part in data collection, group discussions & recom-mendations; targeted area was the North Glasgow Canal corridor	Fauna and flora Potential for area to be so much more Place to feel good Cycling and walking paths away from traffic	Lack of maintenance (grass cutting on verges, derelict and vacant land) Rubbish and vandalism Regulations that prevent use for camping Safety issues	Children made an animated film describing their data-derived findings & recommendations that was shown to their school, local authorities, canals authorities and governmental political groups Data and film were discussed and debated during Science Day at three schools and further data collected Results integrated within a larger body of evidence and have been shared with the Scottish Government cross-political party commit-tee on waterways and University network for the United Nations 26th world summit on climate change occurring in Glasgow Results contributed to the building of a new multi-usage game space for youth near the North Glasgow Canal which allows youth to play football, basketball, hockey and other group sports in a safe environment. A new bridge also is being erected linking three communities previously physically separated by the canals.
**Inter-Generational Youth Projects**				
Gilroy, CA (USA) Safe Routes to Elementary School (SRTS) [61]	Prospective controlled comparison design involving 2 schools; parents of ethnically diverse elementary school children (grades 6 to 8) from Gilroy, CA (total city pop. = 53,231) N = 6 parents ages 46–49 yrs. (83% women) in School A participated	Crossing guards Presence of sidewalks Pedestrian signals Trails available in some areas	Concerns with traffic flow Sidewalk issues Problematic intersection; other unsafe crossings Speeding by parents near school site Other unsafe driver behaviors (e.g., blocking crosswalks) No bike helmets	13 stakeholders from 7 different community sectors engaged with citizen scientists in active solution building School A engaged in twice as many Safe Routes activities (eight) as School B (control; four) across the school year Independently collected tallies of walking/biking to school indicated a six-fold increase in School A & a slight decrease in School B; between-school end-of-yr. walking/biking rates were twice as high in School A vs. School B (*p* < 0.001) During the following year, School A had a three-fold greater number of Safe Routes activities than School B
North Fair Oaks, CA (USA) Neighborhood walkability and security across generations [79]	Assessment of neighborhood built-environment features that help or hinder physical activity (N = 10 adults, mean age 71.3 ± 6.5 yrs.; and 10 adolescent, low-income Latinx; mean age 12.8 ± 0.6 yrs.); 60% of adolescents & all adults female	Having attractive destinations and amenities to visit The aesthetic ‘feel’ of the neighborhood Trees to help reduce pollution Benefit of local school & pediatric clinic to community Covered bus stop to shield from rain	Trash Poor quality sidewalks Personal safety/crime Graffiti Structural impediments to walking, including dangerous intersections	Community-driven solutions included: Trash—Local refuse dept. representative instructed residents on reporting illegal neighborhood dumping & information about trash pick-up days for larger items (e.g., discarded furniture); informed formation of an executive committee of local decision makers to address illegal dumping/trash issue Developed a Bilingual Community Resource Guide with contact details for relevant service providers Personal safety—instructed on forming a neighborhood watch association & working with the city to learn how to fill out forms, start a petition, initiate local action Sidewalks—reporting of unsafe sidewalks to local Department of Public Works Disseminated results through local article featuring teen & older adult participants, & through a national Latino health website
Cuernavaca, Mexico Supporting intergenerational active living across socio-economic strata [78]	Tested acceptability and feasibility of using *Our Voice* method to assess walkability environments in four Mexican neighbor-hoods stratified by socioeconomic status & walkability; (N = 32 adults, mean age = 57.3 ± 8.7 yrs., 9 adolescents mean age = 13.3 ± 1.6 yrs.)	Presence of parks or recreational facilities Having destinations to visit	Poor sidewalk quality Presence of trash Negative street characteristics Unpleasant aesthetics (e.g., graffiti) Feeling unsafe Unleashed dogs Limited disabled access Lack of crosswalks Poor quality of parks and recreational facilities	Creation of a neighborhood committee and campaign to encourage neighbors to use leashes and clean up after their dogs Adults and adolescents discussed and built consensus on acceptable forms of public art, as opposed to uncontrolled graffiti Neighborhood watch programs to combat crime Strategies identified to promote increased social cohesion in the neighborhood Few communication pathways noted between residents and local policy makers & stakeholders
Los Altos, CA USA Multi-sectoral impacts of Pop-Up Parks in an urban setting [80]	Multi-generational community residents (N = 9), with four <17 yrs. old; 88% female; collected data before, during & after pop-up park availability	Pop-up park resulted in more people in location, which added energy to the downtown area It attracted people to local businesses It attracted all age groups Enjoyment of activities at the park Brought positive aesthetics to the area	Without the pop-up park, few people in the area, fewer things to do for different age groups, especially youth; fewer interesting things to see	The information collected was combined with other types of information (surveys, observational data collection) and presented to the local City Council by participating researchers The City Council approved continuation of the pop-up park City sales tax data indicated increases in year-on-year sales tax revenue in the financial quarter in which the parks were in place
**College-Age/Young Adult Projects**				
Palo Alto, CA USA Addressing gender-based violence on college campuses [68]	Feasibility study translating *Our Voice* model to gender-based violence field; focus on campus social spaces creating comfortable or uncomfortable contexts; Participants = 10 under-graduate women ages 18–20 yrs.	Greek life practices that support safety (e.g., ID check to enter) Bathrooms used by women only Gender-balanced classes Gender balance at on-campus events	Noisy/raucous Greek/fraternity parties Gender discomfort created by gender-neutral, shared gender toilets Workspaces where men predominate Large numbers of men at college events daunting Gyms can be intimidating for women	Proposed strategies: (i) modifications to gender spaces, particularly bathrooms in dorms; (ii) change in party-dynamics (e.g., increasing nonfraternity-based parties); (iii) more education about healthy masculinity; (iv) allow/encourage sororities & mixed-gender housing groups to host all-campus parties; (v) include gender dynamics in course evaluation forms; (vi) hire more women professors & teaching assistants Identified decision-makers to help facilitate & implement changes
Auckland, NZ Student voices: What features of the university campus environment influence physical activity and eating habits in University students? [70]	Ethnically diverse university students (aged 17–50 years) across 4 university campus sites in Auckland (locations: North, South, City, Millennium) N = 81 (55 F; 26 M) who completed campus walks; 21 (19 F; 2 M) participated in action groups (n = 3 groups)	Sports facilities & equipment are freely available Natural settings and specialty spaces encourage PA and mental wellbeing Gyms contribute to vibrancy of place & are inexpensive	Lack or limited access to affordable healthy food choices onsite Vending machines with many unhealthy choices Campus isolation from closest supermarket due to perceived distance to walk during breaks Restricted gym times Non-functional green space could be used for activity	The next phase of this research includes presenting these findings to the student union and senior leadership team of the university, evaluating the changes that this type of approach can bring to the university environment and empowering the student community to advocate for change, with the ultimate goal of improving onsite campus features in terms of physical activity and eating habits
Palo Alto, CA USA Promoting gender equity on college campuses [81]	Assessed access to single-occupant all-gender restrooms on Stanford Univ. main campus, Medical School, School of Edu-cation & Graduate Busi-ness School by staff, undergraduate & graduate students, community members (N = 23; ages 18–66, mean age 33 years)	A number of bathrooms on main campus had already been converted Clear all-gender access signage in main campus areas open to public	Many bathrooms dirty or not stocked with supplies (toilet paper, towels, etc.) Some bathrooms inaccessible (i.e., behind locked doors, on second floor of buildings with no elevators) Many unconverted bathrooms in peripheral parts of campus	Participants reported increased interest in learning more about state law regarding all-gender restroom access Increased feelings of community support and collective efficacy among LGBTQ+ participants Data collected on 40 buildings and 200 bathrooms on campus, which were shared with campus building managers and university administrative bathroom access working group to facilitate campus changes
Palo Alto, CA USA Enhancing mental health among graduate students, including during COVID-19 pandemic [72]	Graduate students (N = 7) representing PhD, masters and professional programs (29% women)	Good amount of campus green space Recreational offerings	Lack of structured space that can be used specifically for non-academic, stress-reducing activities	Article published in the university student online newspaper described results & recommendations, including increasing campus spaces for non-academic activities, e.g., designated “no-homework” spaces for crafts, games, & other relaxing activities; free outdoor, socially spaced music activities; more outdoor spaces suitable for solitary activities (e.g., reading, drawing); more outdoor fitness equipment; form “social bubbles” to lessen psychological burdens of the pandemic [82]

### 3.7. User Experiences with the Discovery Tool App and Our Voice Process

Similar to other age groups [29], the vast majority of users and researchers across the diversity of projects described in this article reported generally positive experiences with the Discovery Tool mobile app, which has been developed to run on a variety of mobile devices using Apple iOS and Android operating systems. While over time some participants have faced challenges around connectivity and data upload, the developer team at Stanford generally has been able to troubleshoot and correct problems in a timely manner, without unduly affecting project data collection. Another strength of the Discovery Tool app is that it can be readily translated into other languages. Currently the app is available in 13 languages, and this number will continue to increase as additional global communities are added. In addition, participants have noted the utility of combining both visual (photos) and descriptive information (through using either the audio-recording or texting functions offered in the app) on their local environmental discovery walks. This multi-faceted data capture provides a fuller picture of the lived experience in a given community, going beyond simple photos that can easily be misunderstood without a narration of why the photo was taken. While the mobile app continues to be updated based on user experiences and feedback, the objective of maintaining the app’s overall simplicity of use has been preserved to ensure that diverse groups of residents can successfully use it irrespective of technology literacy or educational level. Although many youth and young adults tend to have greater overall familiarity with and interest in using such mobile technologies relative to other age groups, we have worked to ensure that the app remains easy-to-use and accessible to diverse groups, including those with less overall technology literacy [29].

**Figure 5:**
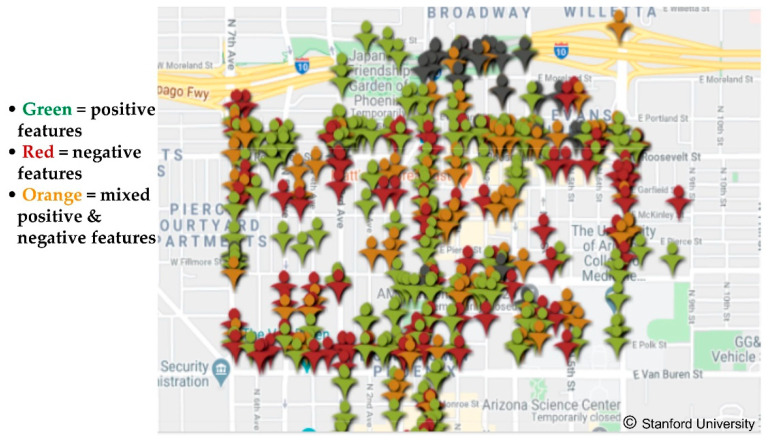
Example of discovery tool geocoded citizen science data grouped by location to identify community “hot spots”.

The Discovery Tool Data Portal houses all of the anonymized Discovery Tool data uploaded to the secure Stanford server by *Our Voice* project participants. The Portal offers an increasing array of options for visualization, mapping, and use of citizen scientist-generated data to compel local action. Figure 5 shows the Portal’s “hotspot” function, which aggregates data gathered by groups of citizen scientists and provides a quick glance at specific areas with a higher density of barriers vs. assets for the target local issue. The Portal features also allow for mapping overlays and integration with other platforms, such as Google Earth and Google Street View, which can enable a more immersive view of the citizen scientists’ collective data (see example at https://www.visibleghosts.com/methodology).

As noted elsewhere [29], the Discovery Tool app is embedded in a constellation of project support tools that enable ongoing project management and coordination across the four steps of the *Our Voice* model. These include an online project management dashboard, user guides for *Our Voice* technologies, community meeting facilitation manuals, action planning templates, and resources for advocacy training and similar activities. The dashboard provides a place where project managers and facilitators can record and track all project activities and outcomes from which project reports can be developed. All project coordinators are invited to participate in the *Our* Voice Global Research Network, which promotes cross-project collaborations, publications, and knowledge exchanges. For those *Our Voice* studies initiated and managed by other universities or organizations and covered by their own human subjects’ Institutional Review Boards (IRBs), participant anonymity is preserved with respect to the Stanford *Our Voice* team managing the data. In those cases, only individuals serving as local project managers and site facilitators know the identities of their own project participants. For studies initiated and/or managed by the Stanford *Our Voice* team, participant confidentiality is protected under Stanford IRB protocol #45330.

The *Our Voice* Global Network has been utilized to facilitate information exchanges related to sharing core measures that can be used across projects to generate cross-cultural and inter-generational learnings [44]. For example, methods for assessing important person-level outcomes such as personal and collective efficacy, agency, and community engagement have been shared among Network members, and have been found to be sensitive to change among youth and adults participating in this type of citizen science research [42,79]. Network members also have provided insights with respect to the varied challenges that can occur with this type of participatory action research. Among such challenges are citizen scientist and community organization recruitment; methods for enhancing continued resident and community partner engagement over time; and applications of the most rigorous study designs and methods available within the context of this type of community-based mixed-methods research paradigm. Examples of such designs are natural experiments [83] and experimental and quasi-experimental pre-posttest comparison group designs [61]. It also is important to ensure clear communication and understandings around who is responsible for implementing the action steps developed during the final steps of the model. This may be an especially important issue when youth and young adults are the citizen scientists and, particularly in the young adult age group, the major drivers of the research project. Given that youth and young adults spend a transitory period of time in different school settings, a formal “transition” plan across school grades or levels may be helpful. This is what occurred in the northern California Safe Routes to School (SRTS) middle school project, where the middle school citizen scientists proactively trained members of the incoming student class to take over portions of the school’s SRTS activities so momentum would not be lost [61].

To facilitate ongoing dissemination of the *Our Voice* method, a brief overview of this citizen science method is available through an online video titled Citizen Science for Health Equity (see Supplementary Materials section). 

## 4. Discussion

Developing strategies for furthering progress towards health equity has become increasingly important in light of the growing health disparities found in many communities and nations around the world [6,27]. Such approaches need to take aim at the social, environmental, cultural and economic conditions impacting the ability of communities to ensure that their residents can live full and healthy lives. Methods for advancing health equity often have focused on multi-sector collaborations with decision-makers and institutional stakeholders (i.e., “top-down” approaches). While the utility of such methods has been noted, “bottom-up” approaches that actively engage residents themselves in documenting as well as participating in changing iatrogenic conditions have been increasingly recognized as additional potentially powerful tools for fostering more equitable community contexts. The group of “by the people” *Our Voice* citizen science projects described adds to the growing literature on youth-engaged participatory action research aimed at fostering more equitable and healthier communities [25,53]. This literature underscores the promise of engaging youth and young adults as active participants as well as potential drivers of positive local community change that can reap benefits for both the participants themselves and their communities. In this way, youth can be viewed as an underutilized resource that can and arguably should be engaged in changing relevant physical and social environments to advance health equity and environmental sustainability. The projects described in this article that included individuals as young as 9 years old indicate that such citizen science/participatory action activities can begin at a relatively early age, with potential benefits with respect to civic engagement accruing across this key developmental period and beyond. The inter-generational projects discussed also indicate that such activities may be a useful way for youth to engage in positive consensus-building interactions with older generations, furthering mutual understandings concerning what may be good for the community as a whole [78,79]. The array of projects presented, which represented youth and young adult populations across the socioeconomic continuum, support the robustness and perceived relevance of this type of systematic yet accessible form of participatory action research in this age group. The *Our Voice* model was specifically developed to be accessible, engaging, and impactful for disadvantaged and marginalized communities which traditionally have lacked a direct means for allowing their voices to be heard as part of local decision-making [84]. Similar acceptance of this participatory action model has been found among geographically and socioeconomically diverse groups of adults and older adults [29,42].

The issues being tackled by young adults, in particular, were varied and far-ranging, including built environment as well as challenging social issues (e.g., gender-based violence). A strength of this approach has been its focus on current, compelling issues in residents’ own local environments and communities, which can enhance youth engagement and commitment. The lack of focus on locally relevant issues that often occurs in youth-focused citizen science aimed at environmental science and conservation has been noted to be a potentially salient issue, given that in these latter fields citizen science targets may be locations and issues removed from young people’s local neighborhoods or communities [23]. This may in turn may make it harder for some youth to maintain engagement over time.

As reflected in this article, educational environments provide an excellent setting for addressing the school environment itself, which can have potentially positive consequences for educational attainment and learning. Educational environments also can serve as a jumping off point for engaging youth in issues affecting the broader community environment beyond the school walls, as was observed in the projects in Barú, Colombia and Glasgow, Scotland. In contrast, few *Our Voice* projects to date have targeted the home setting, which is a critical environment for physical and mental development as well as for education-based support and learning [85,86].

We also found that, while environmental sustainability and climate stabilization issues were not initially an explicit focus of the citizen science projects being conducted, youth often brought an “environmental justice” perspective to their data collection and interpretation activities [79]. This finding speaks to the relevance and importance of combining these two key issues through a more comprehensive “healthy people, healthy planet” lens, given the growing importance of climate change issues in the minds of today’s youth and young adults. While youth-engaged citizen science has been used extensively in the environmental and conservation fields, it often has focused primarily on data collection, surveillance, and dissemination, without necessarily including formal training in youth-based advocacy and collaborative solution building around local problems facing their own communities [23]. This latter step is a hallmark of the type of “by the people” citizen science described in this article [22]. The clear synergies between local changes that can benefit both people’s health as well as the planet—for example, via active transportation—underscore the importance of expanding the multi-issue focus of community-engaged citizen science [62,63,87].

### 4.1. Limitations

While the collection of first-generation studies described in this article has shown some promising results to date with respect to both participant acceptability and reported changes in local environmental elements and structures, several limitations deserve discussion. They include use of simple pre-posttest designs, short follow-up periods, and small numbers of citizen scientists. Currently, larger scale randomized controlled trials are being conducted testing the causal pathways through which such interventions can impact change, along with initial and longer-term effects of the *Our Voice* methods in promoting health behaviors such as regular physical activity in underserved populations. Such studies will help to shed further light on how changes in local physical and social environments emanating from this method can in turn affect both individual and neighborhood/community level behavioral health outcomes. In addition, recruitment of larger numbers of citizen scientists through different selection methods will help to advance knowledge related to what has been referred to as the “whiches conundrum” [88], i.e., which types of people in which locations and under which sets of circumstances may be most successful and benefit the most from this form of participatory action method. This point notwithstanding, it is important to note that the primary outcomes of this type of research are not at the individual level—where selection bias and other internal validity issues are of paramount importance—but at environmental and policy levels of impact [29]. Given this context, it could be argued that inclusion of the smallest number of residents needed to impact local environmental and policy changes may be the most resource-efficient approach in conducting this type of research. It also should be noted that, given the relatively new application of this citizen science model to youth and young adults, cross-project comparisons currently are in their infancy. An important future objective of the *Our Voice* Network is to undertake systematic comparisons across projects to identify similarities as well as distinctions among targeted populations and locales with respect to model implementation and outcomes.

### 4.2. Future Directions

A useful future aim of the *Our Voice* Global Research Network is to build a robust set of validated measurement tools that will allow data harmonization across *Our Voice* projects, thereby potentially accelerating global insights across countries, populations, and target issues [44]. In addition to methods aimed at capturing changes in local environments and policies, there is increasing interest in understanding how such environmental changes can in turn affect resident and community behaviors and perceptions of importance to health, welfare, vitality and quality of life. It is planned that future projects will include these types of longer-term multi-level impacts. To this end, increasingly rigorous study designs that are being tested in this field include large natural experiments evaluating the multi-level health, social, and environmental impacts of innovative public transit systems on vulnerable populations in South America [83], and pre-post experimental and quasi-experimental comparison-group designs [29,61]. Similarly, the fuller array of multi-faceted effects that can appear over time in such community engagement initiatives are currently being assessed systematically in a growing number of *Our Voice* projects through Ripple Effects Mapping methods [89,90,91]. This participatory technique is aimed specifically at involving citizen scientists and relevant stakeholders in visually mapping program efforts and multi-level impacts over time [91].

Additional future directions for this type of participatory action research include expanding inter-generational projects to facilitate further understandings among different age groups and to potentially increase community-wide impacts; explicitly combining health and environmental sustainability targets to promote increased synergies and impacts across these two key global areas; continuing to explore applications of this adaptive model to other diverse populations, including in areas related to race/ethnicity, gender diversity and cognitive or physical abilities; building on the multi-level data capture capabilities of this citizen science platform through the use of mobile sensors aimed at assessing potentially useful factors at the individual level (e.g., sensors that capture physiological stress and similar variables as residents walk through their local environments [92]), as well as at the environmental level (e.g., portable air quality or noise sensors); and exploring ways in which the unique forms of resident-generated micro-environmental data being captured by this method can complement and extend other types of data platforms, including “big data” sets being generated in epidemiological, computational, environmental, medical, and other scientific fields. The resident-collected data produced through such citizen science methods may add more nuanced and contextually valid perspectives of particular relevance for more vulnerable populations. Finally, an important future direction for this Initiative and the Global Network is to proactively promote cross-country interactions, learnings, and synergies across early-stage researchers as well as the young citizen scientists participating in *Our Voice* research projects. Through harnessing the energy, vitality, and creativity of young people in both of these roles through actively connecting them across diverse cultures and problem areas, heightened synergies may ensue. Facilitating such cross-country relationships at both the resident and researcher levels could help to foster greater understanding between nations and potentially help to decrease inter-cultural divisions.

## 5. Conclusions

The expanding complexity of the challenges facing the world, including the COVID-19 pandemic, has accentuated further a long-pressing need for substantive, mixed-methods research into the nature, prevalence, distribution, and lessening of health disparities. While current quantitative data collection and monitoring efforts aimed at health factors related to COVID-19 and similar challenges will provide critical insights, participatory action models such as *Our Voice* aim to take prevention upstream to the mutable neighborhood and community-level microenvironments that directly impact health behaviors and wellbeing within disadvantaged communities, including amidst the paradigm-shifting local governmental policies (e.g., spatial distancing, sheltering-in-place, wearing of masks) emanating directly from the outbreak. It takes particular aim at building relevant, accessible, and fruitful transactional links between residents and the real-world community contexts in which they live to foster health-enhancing behaviors and social connections that impact both physical and mental health and wellbeing. Harnessing diverse resident insights and perspectives is critical for informing as well as driving more relevant and sustainable solutions as building blocks for healthier communities both now and in the future. Such technology-enabled citizen science models can go beyond knowledge enrichment to build community capacity and support residents from all walks of life as agents of change in advancing health equity in their own locales. In addition, by tracking community-generated activities and outcomes, we will better understand the power of such “bottom-up” resident-community-academic partnerships in promoting change from within communities to prevent or diminish health disparities and potentially foster environmental justice for vulnerable populations.

## Figures and Tables

**Table 2:** Examples of how the *Our Voice* citizen science model can be adapted to a clinical setting.

Participants	Discover	Discuss	Advocate	Change
**Patient and family**	Use Discovery Tool to document factors impacting ability to practice healthy behaviors	In small (remotely connected) groups with other patients and their families, discuss, organize and prioritize findings (facilitated by OV team)	Develop action plans and present to clinicians for discussion	Individual, social, environmental and policy changes are measured
**Clinician** (individual)	Obtain a better understanding of their patients’ neighborhoods	Learn about positive and negative opportunities related to health behaviors (e.g., physical activity, social distancing for COVID-19, local park access & use)	Example: Signing a neighborhood letter for a patient or patient group advocating for a feasible neighbor-hood change	Stronger relationships with patients and community, enriched by contextual knowledge
**Clinic** (group)	Develop interactive neighborhood maps and share in clinic setting and/or online	Highlight areas of opportunities/concern and how the community can help	Example: Clinic helps to advocate for larger collective change at local community or gov-ernmental levels	Clinic becomes a key community hub for citizen science and positive “up-stream” activities

## Data Availability

The data presented in this study are available on request from the corresponding author. The data are not publicly available due to privacy issues.

## Supplementary Materials

Video S1: Our Voice: Citizen Science for Health Equity https://www.youtube.com/watch?v=sYcYXh51Bl0.
